# Association of body mass index with rapid eye movement sleep behavior disorder in Parkinson’s disease

**DOI:** 10.3389/fneur.2024.1388131

**Published:** 2024-05-23

**Authors:** Si-Chun Gu, Xiao-Lei Yuan, Ping Yin, Yuan-Yuan Li, Chang-De Wang, Min-Jue Gu, Li-Min Xu, Chen Gao, You Wu, Yu-Qing Hu, Can-Xing Yuan, Yang Cao, Qing Ye

**Affiliations:** ^1^Longhua Hospital, Shanghai University of Traditional Chinese Medicine, Shanghai, China; ^2^Ruijin Hospital, Shanghai Jiao Tong University School of Medicine, Shanghai, China; ^3^Shanghai TCM-integrated Hospital, Shanghai University of Traditional Chinese Medicine, Shanghai, China; ^4^Yueyang Hospital of Integrated Traditional Chinese and Western Medicine, Shanghai University of Traditional Chinese Medicine, Shanghai, China

**Keywords:** body mass index, rapid eye movement sleep behavior disorder, Parkinson’s disease, Parkinson’s progression markers initiative, restricted cubic spline

## Abstract

**Background:**

The association between body mass index (BMI) and rapid eye-movement (REM) sleep-related behavioral disorder (RBD) in Parkinson’s disease (PD) remains unknown. Our study was to investigate the association of BMI with RBD in PD patients.

**Methods:**

In this cross-sectional study, a total of 1,115 PD participants were enrolled from Parkinson’s Progression Markers Initiative (PPMI) database. BMI was calculated as weight divided by height squared. RBD was defined as the RBD questionnaire (RBDSQ) score with the cutoff of 5 or more assessed. Univariable and multivariable logistic regression models were performed to examine the associations between BMI and the prevalence of RBD. Non-linear correlations were explored with use of restricted cubic spline (RCS) analysis. And the inflection point was determined by the two-line piecewise linear models.

**Results:**

We identified 426 (38.2%) RBD. The proportion of underweight, normal, overweight and obese was 2.61, 36.59, 40.36, and 20.44%, respectively. In the multivariate logistic regression model with full adjustment for confounding variables, obese individuals had an odds ratio of 1.77 (95% confidence interval: 1.21 to 2.59) with RBD compared with those of normal weight. In the RCS models with three knots, BMI showed a non-linear association with RBD. The turning points of BMI estimated from piecewise linear models were of 28.16 kg/m^2^, 28.10 kg/m^2^, and 28.23 kg/m^2^ derived from univariable and multivariable adjusted logistic regression models. The effect modification by depression on the association between BMI and RBD in PD was also found in this study. Furthermore, the sensitivity analyses linked with cognition, education, and ethnic groups indicated the robustness of our results.

**Conclusion:**

The current study found a significant dose–response association between BMI and RBD with a depression-based difference in the impact of BMI on RBD in PD patients.

## 1 Introduction

Body mass index (BMI) is a widely used measure of relative body weight and is considered the gold standard of general nutritional status (1). Both high and low BMI are directly related to health risks including hypertension, type 2 diabetes, cardiovascular disease, and certain cancers, and can result in detrimental health outcomes. A change in BMI is considered an important outcome in monitoring health and well-being (2, 3). BMI has been found to be associated with function in several neurodegenerative diseases. In patients with amyotrophic lateral sclerosis, a BMI < 18.5 kg/m^2^ is associated with reduced survival, while a BMI of 30–35 kg/m^2^ is associated with increased survival (4). In patients with dementia, a low BMI is associated with reduced survival and serves as an independent predictor of mortality, regardless of cognitive impairment severity (5).

Parkinson’s disease (PD) is a neurological degenerative disease characterized by motor symptoms including rigidity, tremor, bradykinesia, and postural instability, along with a wide range of non-motor symptoms, including sleep disturbances, autonomic dysfunction, neuropsychiatric disorders, and cognitive impairment (6). The combination of the heterogeneous symptomatology mentioned above directly threatens the ability of individuals with PD to live independently and imposes a significant economic and global burden (7, 8).

Given the high prevalence of weight variation in PD, increasing attention is being paid to investigate the role of BMI in PD. For the diagnosis of PD, several studies have associated obesity with a higher risk of developing PD. Conversely, patients with PD are consistently reported to have lower body weight compared to healthy controls (9). When considering survival in PD, there is a significant inverse dose–response relationship between BMI and mortality. BMI > 23 kg/m^2^ contributes to extended survival rates, while BMI < 18.5 kg/m^2^ is linked with poor survival (10). The association between BMI and motor symptoms of PD has been explored, that is decreasing-BMI is associated with worse scores over time in UPDRS motor scores, whereas increasing-BMI is associated with better UPDRS motor scores (11). Although the biological mechanisms have not been identified, potential contributors may include perturbation of hypothalamic metabolic regulation, gastrointestinal dysfunction, and alteration of energy expenditure and food intake (12).

However, to the best of our knowledge, the effects of BMI on PD non-motor symptoms have not been previously studied. As the most common disabling non-motor symptom of PD, the prevalence of rapid eye-movement (REM) sleep-related behavioral disorder (RBD) is around 20 to 50% in PD patients (13). RBD is characterized by a loss of muscle atonia during REM sleep, leading to dream enactment behaviors that are frequently injurious to patients and their partners (14, 15). Previous studies have clearly observed U-shaped and inverse U-shaped relationships between sleep duration and BMI (16). However, the impact of BMI on RBD in PD has not been fully clarified, and understanding is crucial for improving outcomes related to non-motor symptoms in patients with PD. Therefore, the objective of this study was to investigate the association between BMI and RBD in patients with PD using a large population-based dataset.

## 2 Methods

### 2.1 Study design

Study data used in the present study were obtained from the Parkinson’s Progression Markers Initiative (PPMI) database.^1^ PPMI is an ongoing observational, international, multicentre cohort study aiming to identify blood-based, genetic, spinal fluid, and imaging biomarkers of Parkinson’s disease (PD) progression with longitudinal follow-up in a large cohort. The aims and methodology of PPMI study have been published elsewhere (17). Study protocol and manuals and are available online. The study was approved by the Institutional Review Board at each site, and all participants provided written informed consent.

**Figure 1:**
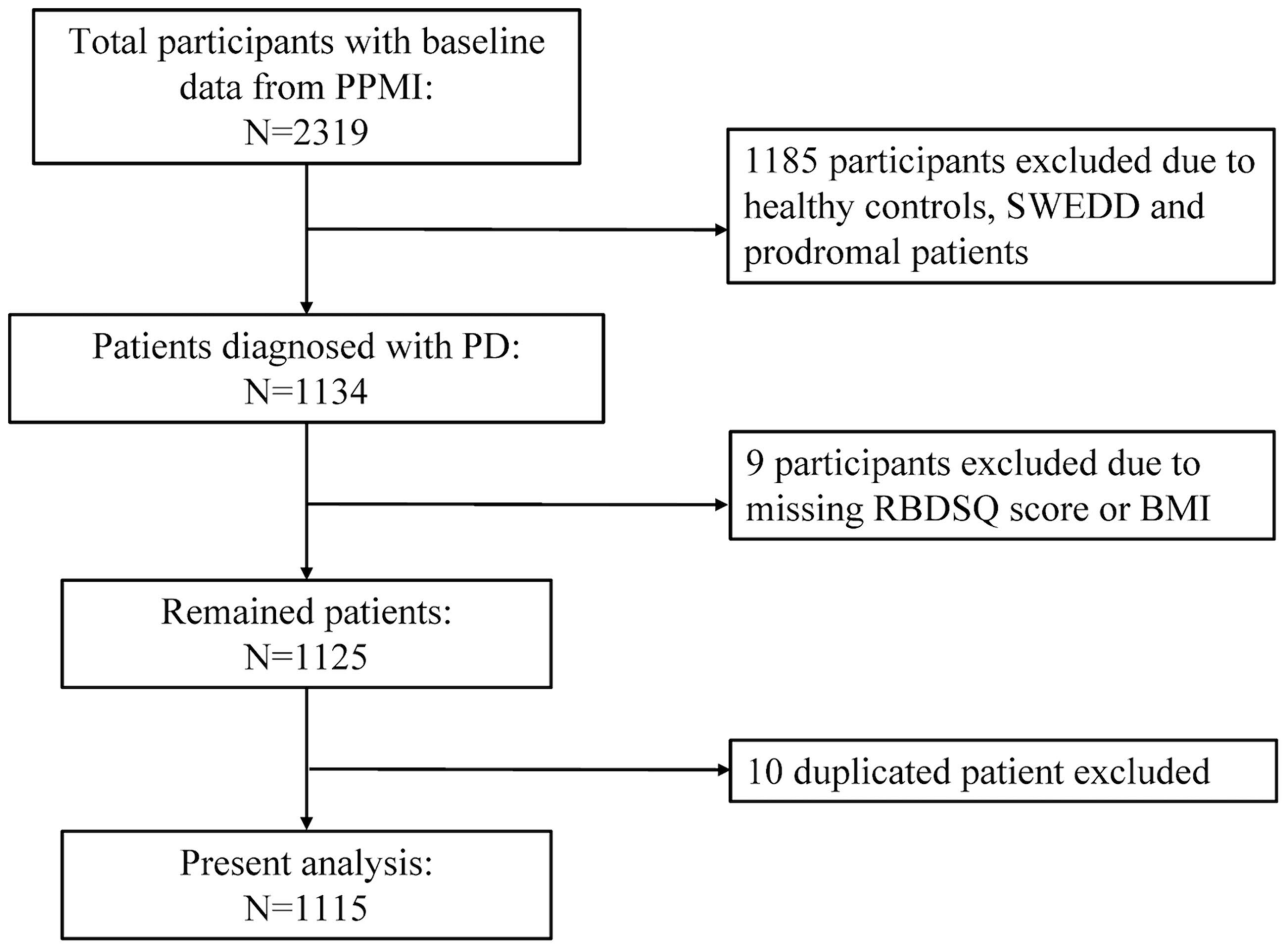
Flow chart of the study population. PPMI, Parkinson’s Progression Markers Initiative; PD, Parkinson’s disease; SWEDD, scans without evidence of dopaminergic deficit; RBDSQ, rapid eye movement (REM) sleep behavior disorder questionnaire; BMI, body mass index.

For this study, we utilized the dataset of PPMI from 33 participating outpatient PD treatment centers worldwide on the basis of inclusion and exclusion criteria previously published. All the methods were performed in accordance with relevant institutional guidelines and regulations. The Strengthening the Reporting of Observational studies in Epidemiology (STROBE) guideline was included in Supplementary material. Figure 1 illustrated the selection process of our study. Participants who were diagnosed as idiopathic PD, with Rapid eye movement (REM) sleep behavior disorder questionnaire (RBDSQ) score and BMI data were included as study population. Healthy controls, scans without evidence of dopaminergic deficit (SWEDD) patients, prodromal patients, duplicated participants, individuals with missing RBDSQ score or BMI data were excluded from this analysis. In total, 1,115 patients with complete information, enrolled between November 2010 and June 2023, were included in our analyses. The data were downloaded on September 15, 2023.

### 2.2 Exposure and outcome

Exposure was assigned as BMI. Anthropometric data, including height and weight, were obtained from PPMI data. BMI was calculated as weight divided by height squared [weight (kg)/height (m^2^)], and then classified into 4 WHO categories, including underweight (BMI < 18.5 kg/m^2^), normal weight (18.5 ≤ BMI < 25.0 kg/m^2^), overweight (25 ≤ BMI < 30 kg/m^2^) and obese (BMI ≥ 30 kg/m^2^) categories (18, 19).

Outcome was assigned as REM-sleep behavior disorder (RBD). The 10-item RBDSQ has been validated in PD patients and demonstrates good accuracy in identifying RBD (20). Items 1 to 4 of the RBDSQ assess the frequency and content of dreams, as well as their relationship to nocturnal movements and behavior. Item 5 asks about self-injuries and injuries to the bed partner. Item 6 consists of four subitems specifically assessing nocturnal motor behavior, including questions about nocturnal vocalization, sudden limb movements, complex movements, or items falling from the bed. Items 7 and 8 inquire about nocturnal awakenings. Item 9 focuses on general sleep disturbances, while item 10 pertains to the presence of any neurological disorder. The maximum total score on the RBDSQ is 13 points. Following the definition set by the International Parkinson and Movement Disorders Society (MDS) Task Force, we defined RBD as a RBDSQ score equal to or greater than 5 (20).

### 2.3 Covariates

To assess the potential influence of confounding factors, important covariates were considered on the basis of previous evidence and applicability to PPMI: age, sex, PD duration, depression (measured by the 15-item Geriatric Depression Scale [GDS-15] score in PPMI), levodopa equivalent daily dose (LEDD), hypertension (defined as self-reported hypertension, systolic blood pressure ≥ 140 mmHg, diastolic blood pressure ≥ 90 mmHg, or use of antihypertensive drugs), and MDS Unified Parkinson’s Disease Rating Scale (MDS-UPDRS) score (21). All participants in the PPMI study underwent the standard test battery of assessments. In addition to the covariates mentioned above, sociodemographic characteristics and clinical battery relevant to this study including education, Hoehn & Yahr stage, serum uric acid, autonomic function assessed by the Scale for Outcomes for Parkinson’s Disease-autonomic function (SCOPA-AUT) score, anxiety assessed by State-Trait Anxiety Inventory (STAI) score, and daily living quality assessed by Modified Schwab and England Activities of Daily Living Scale (MSEADL) were adjusted as potential confounders in the models. Although PPMI collected an array of cerebrospinal fluid (CSF) biomarkers, these measures were only available for a small subset of participants and thus were not included in this study.

### 2.4 Statistical analysis

Summary statistics were performed and tested for normality (Shapiro–Wilk). Continuous data were presented as median (interquartile range [IQR]) or mean ± standard deviation (mean ± SD), with categorical data presented as proportion and number (*N* [%]) as appropriate. Group comparisons were analyzed with use of Student’s *t* tests or Wilcoxon’s rank-sum tests for continuous data and Chi-square tests or Fisher’s exact tests for categorical data. Data were more than 99% complete. The remaining missing values were imputed by multivariable chained imputation with fully conditional specification, and imputed and reported results were similar (22). All statistical tests were two-sided and the level of significance was set at 0.05.

Participants were divided into two groups based on whether they had RBD. The relationship between BMI and RBD was examined using logistic regression models. The adjustment was accomplished via 3 models: (1) model 1, without any covariate adjustment; (2) model 2, adjusted for age, sex, PD duration, GDS-15 score, LEDD, hypertension, depression, and MDS-UPDRS I, II and III scores; (3) model 3, additionally adjusted for education level, serum uric acid, Hoehn & Yahr stage, STAI, SCOPA-AUT, and MSEADL scores as covariates. The results were presented as odds ratios (OR) with corresponding 95% confidence intervals (CI).

Restricted cubic spline (RCS) analysis was also performed to examine the association of between BMI and RBD based on univariable and multivariable adjusted logistic regression models (23). To balance best fit and overfitting in the main splines, the number of knots, between three and seven, was chosen as the lowest value for the Akaike information criterion. If the difference in the number of knots was within two for different models, the lowest number of knots was selected (24). The same number of knots from the main splines was also applied for stratified analyses to allow direct comparison of overall and stratified analyses, including test of interaction. We tested for potential non-linearity by using a likelihood ratio test comparing the model with only a linear term against the model with linear and cubic spline terms. Piecewise-linear models were then fitted to quantify the association between BMI and RBD. If evidence of non-linearity was found, a two-line piecewise linear model with a single change point was estimated by trying all possible values for the change point and selecting the value with the highest likelihood among those considered, while accounting for covariates.

We fitted interactions to investigate effect modification by depression (with depression, without depression, GDS-15 ≥ 5 or not), motor subtype [tremor dominate (TD), non-TD including postural instability/gait disorder (PIGD) or Indeterminate], sex (male, female), and hypertension (yes, no) (25–27). Due to the nonlinear association between BMI and RBD in the whole participants, we used continuous BMI and the quadratic term BMI^2^ in multivariable adjusted logistic regression models (model 3) to allow for the nonlinearity during the interaction analyses. The first model to test for the depression-by-BMI interaction allowed for interaction with both the linear and quadratic terms of BMI (28).


$$\begin{array}{l} \mathrm{Model}\;A:\mathrm{RBD}=\mathrm{BMI}+{\mathrm{BMI}}^{2}+\mathrm{depression}+\mathrm{BMI}\times \mathrm{depression}+\\ \;\;\;\;\;\;\;\;\;\;\;\;\;\;\;\;\;\;\;\;\;\;\;\;\;{\mathrm{BMI}}^{2}\times \mathrm{depression}+\mathrm{other covariates} \end{array}$$

In the absence of interaction with the quadratic term, the model was then simplified to only allow for interaction with the linear term.


$$\begin{array}{l} \mathrm{Model}\;B:\mathrm{CVD}=\mathrm{BMI}+{\mathrm{BMI}}^{2}+\mathrm{subtype}+\mathrm{BMI}\times \\ \;\;\;\;\;\;\;\;\;\;\;\;\;\;\;\;\;\;\;\;\;\;\;\;\;\mathrm{depression}+\mathrm{other covariates} \end{array}$$

The significance of the interaction was determined based on the highest level interaction term in the models, and here, lack of interaction was inferred when neither BMI^2^ × depression (Model A) nor BMI × depression (Model B) were significant at the 5% level. Interactions by subtype, hypertension, and sex were examined in the corresponding manner, replacing “depression” above with hypertension, sex or subtype as relevant.

To assess the robustness of the results, we additionally applied three sensitivity analyses. First, we examined the shape of BMI-RBD relation after excluding individuals who had a Montreal Cognitive Assessment (MOCA) score less than 26 as the definition of cognitive impairment (29). Second, we restricted the analysis to individuals in the first and second categories of education. Third, we performed the analysis after excluding other ethnic groups. All data were analyzed using R (version 4.0.2).

## 3 Results

Of 1,115 participants included in the study, we identified 426 (38.2%) RBD. Table 1 summarized the characteristics of participants according to BMI categories. The proportion of underweight, normal, overweight and obese was 2.61, 36.59, 40.36, and 20.44%, respectively. The median BMI was 26.1, and the median age was 63.6 years. Within 4 BMI categories, significant differences were observed regarding the proportion of RBD, RBDSQ score, sex, education status, MOCA score, GDS-15 score, SCOPA-AUT score, proportion of hypertension, and serum uric acid. The individuals with higher BMI tended to have higher RBDSQ score, as well as a higher proportion of RBD. The proportion of RBD decreased with higher BMI. The proportion of male decreased with higher BMI. The individuals with lower BMI tended to have a lower proportion of the first category of education (<13 years) and a higher proportion of the second category of education (13–23 years). Underweight individuals had higher MOCA score, GDS-15 score, and SCOPA-AUT score than others. Serum uric acid increased with higher BMI, with the median of 4.1 mg/dL in underweight individuals, 4.5 mg/dL in normal weight individuals, 5.4 mg/dL in overweight individuals, and 5.5 mg/dL in obese individuals. Moreover, a positive association was observed between hypertension and BMI. 17.2% of underweight individuals had hypertension, whereas 49.6% of obese individuals had hypertension.

**Table 1:** Characteristics of study population according to BMI measure by WHO BMI category.

	Overall *N* = 1,115	Underweight (<18.5 kg/m^2^) *N* = 29	Normal weight (18·5–24·9 kg/^2^) *N* = 408	Overweight (25·0–29·9 kg/m^2^) *N* = 450	Obese (≥30·0 kg/m^2^) *N* = 228	*p*-value
BMI	26.1 [23.9–29.4]	17.9 [16.9–18.4]	23.3 [21.7–24.2]	27.3 [26.0–28.7]	32.2 [30.9–34.6]	<0.001
Age (years)	63.6 [56.2–69.6]	61.0 [51.5–69.5]	63.2 [54.7–69.3]	64.2 [57.1–70.2]	64.2 [56.8–69.0]	0.207
Sex						<0.001
Male	435 (39.0%)	19 (65.5%)	201 (49.3%)	130 (28.9%)	85 (37.3%)	
Female	680 (61.0%)	10 (34.5%)	207 (50.7%)	320 (71.1%)	143 (62.7%)	
Education (years)	16.0 [14.0–18.0]	16.0 [15.0–18.0]	16.0 [14.0–18.0]	16.0 [14.0–18.0]	16.0 [13.0–18.0]	0.01
Education categories						0.05
<13 years	192 (17.2%)	3 (10.3%)	52 (12.7%)	89 (19.8%)	48 (21.1%)	
13–23 years	904 (81.1%)	26 (89.7%)	347 (85.0%)	354 (78.7%)	177 (77.6%)	
>23 years	19 (1.7%)	0 (0.0%)	9 (2.2%)	7 (1.6%)	3 (1.3%)	
Race						0.379
White	1,043 (93.5%)	28 (96.6%)	385 (94.4%)	415 (92.2%)	215 (94.3%)	
Black	17 (1.5%)	0 (0.0%)	3 (0.7%)	11 (2.4%)	3 (1.3%)	
Asian	14 (1.3%)	1 (3.4%)	7 (1.7%)	5 (1.1%)	1 (0.4%)	
Other	41 (3.7%)	0 (0.0%)	13 (3.2%)	19 (4.2%)	9 (3.9%)	
PD duration (months)	7.8 [3.7–20.6]	8.7 [3.5–14.8]	8.6 [4.2–22.5]	7.1 [3.5–19.1]	8.2 [4.2–22.4]	0.156
LEDD	141.8 ± 317.0	155.1 ± 366.4	169.1 ± 335.6	122.4 ± 292.4	147.0 ± 328.3	0.317
Hoehn & Yahr Stage						0.871
Stage1	377 (33.8%)	10 (34.5%)	139 (34.1%)	152 (33.8%)	76 (33.3%)	
Stage2	713 (63.9%)	18 (62.1%)	261 (64.0%)	290 (64.4%)	144 (63.2%)	
Stage3	25 (2.2%)	1 (3.4%)	8 (2.0%)	8 (1.8%)	8 (3.5%)	
Motor subtype						0.178
TD	742 (66.5%)	17 (58.6%)	266 (65.2%)	294 (65.3%)	165 (72.4%)	
non-TD (PIGD or Indeterminate)	373 (33.5%)	12 (41.4%)	142 (34.8%)	156 (34.7%)	63 (27.6%)	
MDS-UPDRS I score	6.0 [3.0–9.0]	7.0 [3.0–11.0]	5.0 [3.0–9.0]	6.0 [3.0–9.0]	6.0 [3.0–10.0]	0.119
MDS-UPDRS II score	6.0 [3.0–9.0]	7.0 [3.0–11.0]	6.0 [3.0–9.0]	5.0 [2.0–9.0]	5.5 [3.0–10.0]	0.277
MDS-UPDRS III score	20.0 [14.0–27.5]	21.0 [15.0–29.0]	19.5 [14.0–26.0]	20.0 [14.0–27.0]	21.0 [15.0–29.5]	0.228
Total MDS-UPDRS score	33.0 [23.0–43.0]	32.0 [25.0–50.0]	32.5 [23.0–43.0]	32.0 [23.0–42.0]	35.0 [24.0–47.0]	0.085
RBDSQ score	4.0 [2.0–6.0]	3.0 [2.0–6.0]	3.0 [2.0–6.0]	4.0 [2.0–6.0]	4.0 [3.0–7.0]	<0.001
With RBD						0.012
No	689 (61.8%)	17 (58.6%)	270 (66.2%)	281 (62.4%)	121 (53.1%)	
Yes	426 (38.2%)	12 (41.4%)	138 (33.8%)	169 (37.6%)	107 (46.9%)	
MOCA score	26.7 ± 2.7	27.8 ± 2.3	26.5 ± 3.0	26.5 ± 2.8	26.9 ± 2.5	0.005
MSEADL score	92.5 ± 8.0	92.2 ± 7.1	91.8 ± 7.6	93.1 ± 7.6	92.3 ± 8.5	0.224
GDS-15 score	2.0 [1.0–3.5]	2.0 [2.0–5.0]	2.0 [0.0–3.0]	2.0 [0.0–3.0]	2.0 [1.0–4.0]	0.005
STAI score	62.0 [50.0–77.0]	77.0 [58.0–91.0]	62.0 [51.0–77.5]	61.5 [49.0–76.0]	60.0 [49.0–78.0]	0.224
SCOPA-AUT score	9.0 [6.0–14.0]	14.0 [6.0–18.0]	10.0 [6.0–14.0]	9.0 [5.0–14.0]	9.0 [6.0–14.0]	0.005
With hypertension						<0.001
No	655 (58.7%)	24 (82.8%)	264 (64.7%)	252 (56.0%)	115 (50.4%)	
Yes	460 (41.3%)	5 (17.2%)	144 (35.3%)	198 (44.0%)	113 (49.6%)	
Serum Uric Acid (mg/dL)	5.0 [4.2–5.9]	4.1 [3.4–5.4]	4.5 [3.9–5.3]	5.4 [4.5–6.1]	5.5 [4.5–6.4]	<0.001

Data were presented as *N* (%), mean ± SD, or median [IQR]. BMI, body mass index; PD, Parkinson’s disease; LEDD, levodopa equivalent daily dose; MDS-UPDRS, movement disorders society unified Parkinson’s disease rating scale; RBDSQ, rapid eye movement sleep behavior disorder screening questionnaire; GDS-15, 15-item Geriatric depression scale; SCOPA-AUT, scale for outcomes for Parkinson’s disease-autonomic function; STAI, state-trait anxiety inventory; MSEADL, modified Schwab and England activities of daily living; MOCA, Montreal cognitive assessment; *N*, number; IQR, interquartile range; SD, standard deviation.

Table 2 showed the association of BMI and RBD. In the crude logistic regression model, obese individuals had an OR of 1.73 (95% CI 1.24 to 2.41) for RBD compared with normal weight individuals, which indicated that the odds of RBD were increasing for rise in BMI. The strong positive association between BMI and RBD consistently existed in adjusted models. Compared with those of normal weight, obese individuals had ORs of 1.63 (95% CI 1.14 to 2.34) and 1.77 (95% CI 1.21 to 2.59) for RBD in model 2 and model 3.

**Table 2:** The association between baseline BMI and the incidence of RBD in PD.

Model	BMI category	Events/participants	RBD	
			OR (95%CI)	*p* -value
Model 1^a^	Obese	107/228	1.73 (1.24–2.41)	0.001
	Overweight	169/450	1.17 (0.89–1.56)	0.25
	Underweight	12/29	1.38 (0.63–2.95)	0.41
	Normal weight	138/408	Reference	–
Model 2^b^	Obese	107/228	1.63 (1.14–2.34)	0.008
	Overweight	169/450	1.14 (0.84–1.55)	0.39
	Underweight	12/29	1.23 (0.52–2.83)	0.62
	Normal weight	138/408	Reference	–
Model 3^c^	Obese	107/228	1.77 (1.21–2.59)	0.003
	Overweight	169/450	1.17 (0.85–1.60)	0.34
	Underweight	12/29	1.16 (0.48–2.70)	0.74
	Normal weight	138/408	Reference	–

a No covariate was adjusted.

b Age, sex, PD duration, GDS-15 score, LEDD, hypertension, and MDS-UPDRS I, II and III scores were adjusted.

c Education level, serum uric acid, Hoehn & Yahr stage, motor subtype, STAI, SCOPA-AUT, and MSEADL scores were additionally adjusted.

BMI, body mass index; PD, Parkinson’s disease; OR: odds ratio; CI: confidence interval; RBD, rapid eye movement sleep behavior disorder; LEDD, levodopa equivalent daily dose; MDS-UPDRS, movement disorders society unified Parkinson’s disease rating scale; GDS-15, 15-item geriatric depression scale; SCOPA-AUT, scale for outcomes for Parkinson’s disease-autonomic function; STAI, state-trait anxiety inventory; MSEADL, modified Schwab and England activities of daily living.

**Figure 2:**
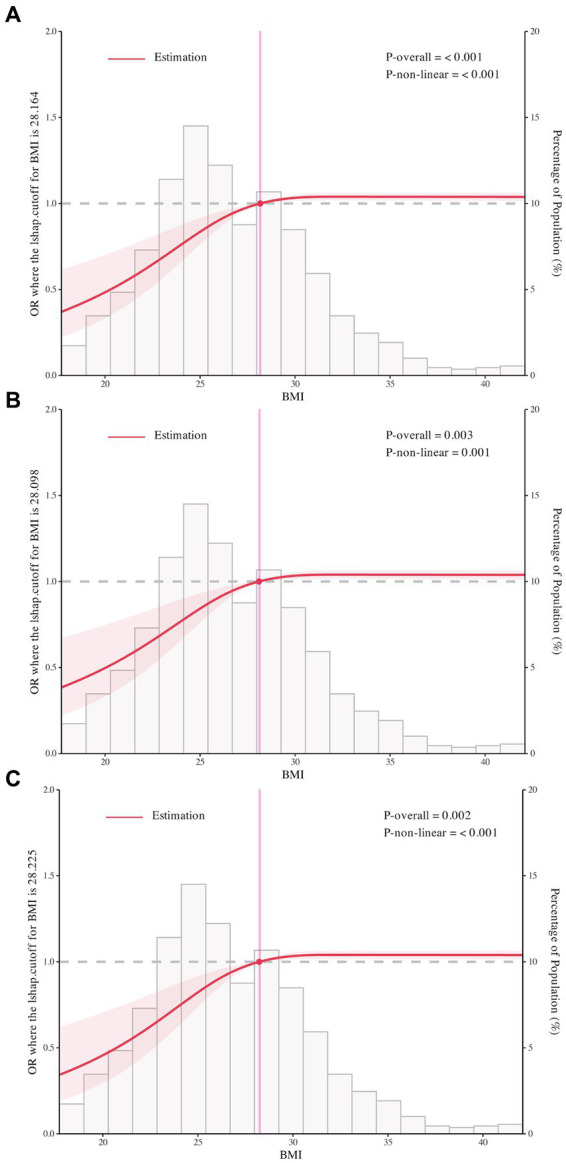
The restricted cubic spline for the association of body mass index with rapid eye movement sleep behavior disorder in Parkinson’s Disease. Odds ratios were indicated by solid lines and 95% confidence interval by shaded areas based on the restricted cubic spline models. For **(A)**, rapid eye movement sleep behavior disorder was not adjusted for any covariate. For **(B,C)**, adjusted factors were consistent with model 2 and model 3. All figures were created with the use of R software (version 3.3.3, https://www.r-project.org/).

We used RCS to flexibly model and visualize the relation of BMI with RBD in PD with three knots (Figures 2A–C). For the odds of RBD, there was evidence of non-linearity with two main patterns seen: we found positive associations below the change points of BMI with the rapid increase of the odds of RBD, whereas there was little evidence of association above the change points of BMI with relative flat trend (*P* for non-linearity <0.001). The change points of BMI estimated from piecewise linear models were of 28.16 kg/m^2^, 28.10 kg/m^2^, and 28.23 kg/m^2^ in univariable and multivariable adjusted logistic regression models (Table 3).

**Table 3:** Estimated change points in the association between BMI and RBD in PD below and above the change point from piecewise two-line models.

	BMI change point (kg/m^2^)	OR per 1 kg/m^2^ BMI increase below change point (95% CI)	OR per 1 kg/m^2^ BMI increase above change point (95% CI)
Model 1^a^	28.16	1.07 (1.01–1.14)	0.99 (0.95–0.9986)
Model 2^b^	28.10	1.07 (1.01–1.15)	1.01 (0.99–1.00)
Model 3^c^	28.23	1.08 (1.003–1.16)	1.00 (0.95–1.00)

a No covariate was adjusted.

b Age, sex, PD duration, GDS-15 score, LEDD, hypertension, and MDS-UPDRS I, II and III scores were adjusted.

c Education level, serum uric acid, Hoehn & Yahr stage, motor subtype, STAI, SCOPA-AUT, and MSEADL scores were additionally adjusted.

BMI, body mass index; PD, Parkinson’s disease; OR: odds ratio; CI: confidence interval; RBD, rapid eye movement sleep behavior disorder; LEDD, levodopa equivalent daily dose; MDS-UPDRS, movement disorders society unified Parkinson’s disease rating scale; GDS-15, 15-item geriatric depression scale; SCOPA-AUT, scale for outcomes for Parkinson’s disease-autonomic function; STAI, state-trait anxiety inventory; MSEADL, modified Schwab and England activities of daily living.

**Figure 3:**
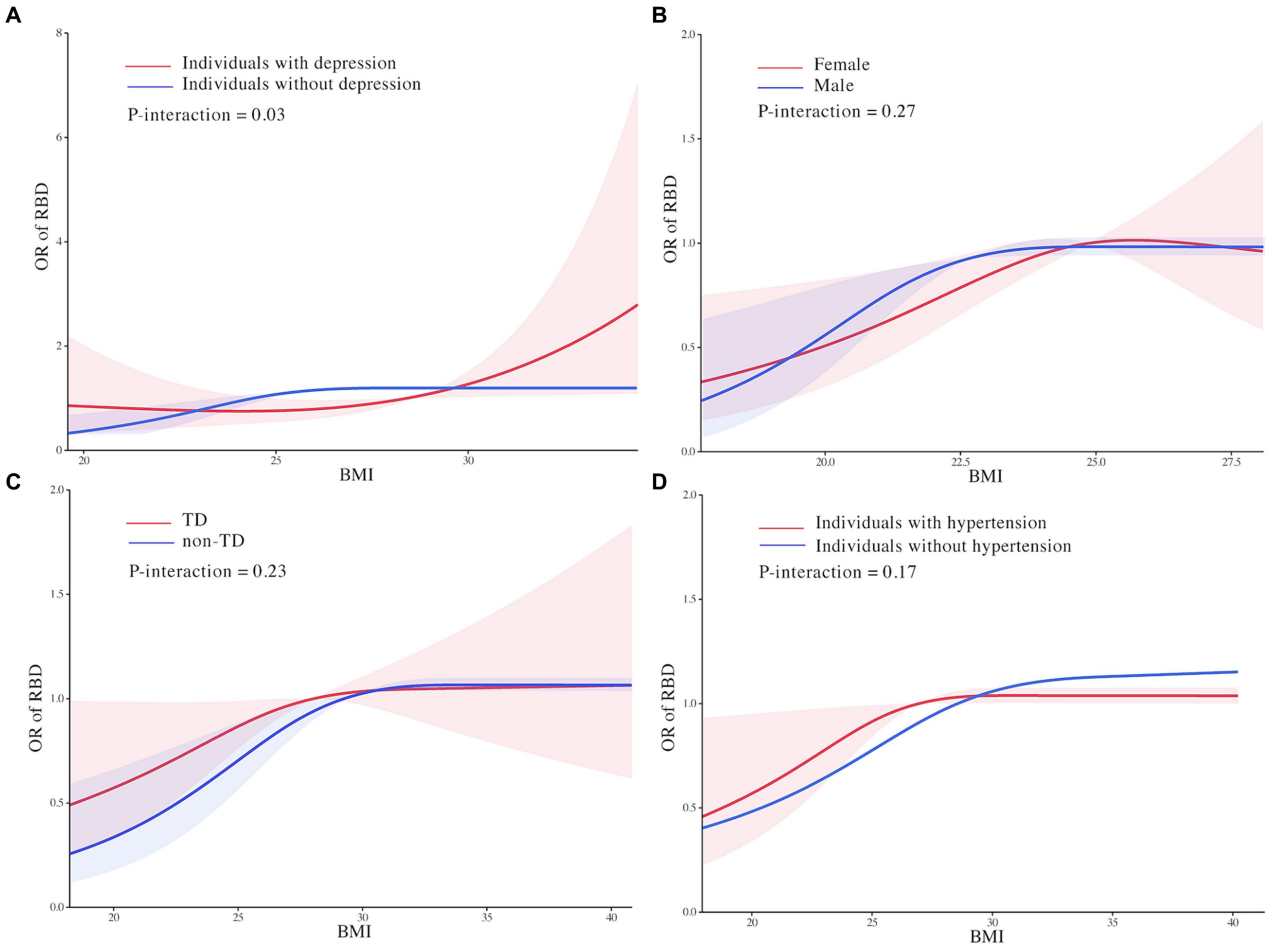
Effect modification of depression **(A)**, motor subtype **(B)**, sex **(C)**, and hypertension **(D)** on the association of body mass index with rapid eye movement sleep behavior disorder in Parkinson’s Disease. Odds ratios were indicated by solid lines and 95% confidence interval by shaded areas based on the restricted cubic spline models. Estimates were adjusted for age, sex, Parkinson’s Disease duration, 15-item Geriatric Depression Scale score, levodopa equivalent daily dose, hypertension, depression, Unified Parkinson’s Disease Rating Scale I, II, and III scores, education level, serum uric acid, Hoehn & Yahr stage, State-Trait Anxiety Inventory score, Scale for Outcomes for Parkinson’s Disease-autonomic function score, and Modified Schwab and England Activities of Daily Living Scale score. All figures were created with the use of R software (version 3.3.3, https://www.r-project.org/).

The effect modification by depression on the association between BMI and RBD in PD was found in this study. In individuals with depression, we found that the relationship between BMI and RBD was linear with the odds of RBD increasing continuously with higher BMI (OR: 1.08, 95% CI 1.002 to 1.18, *P* for non-linearity = 0.15; Figure 3A). In individuals without depression, the threshold effect analysis of BMI on RBD was further performed by the two-piecewise linear regression. The odds of RBD increased rapidly until BMI of 28.31 kg/m^2^ (OR: 1.10, 95% CI: 1.01 to 1.19) with minimal change afterwards (OR: 0.98, 95% CI: 0.93 to 1.00, *P* for non-linearity = 0.001; Figure 3A). We found no evidence of effect modification by sex, motor subtype and hypertension on the association between BMI and RBD in PD (Figures 3B–E; Supplementary Table S1).

The results of sensitivity analyses were similar to the main analysis, when excluding participants with MOCA score less than 26, restricting the analysis to individuals in the first and second categories of education, or excluding other ethnic groups, which indicating the robustness of our results (Supplementary Figure S1).

## 4 Discussion

To our knowledge, this was the first study to investigate the association between BMI and non-motor symptom of PD. In the current study, we examined the association between RBD and BMI among PD patients with use of PPMI cross-sectional data. Our findings showed the significant presence of dose–response relationship between BMI and RBD with a depression-based difference in the impact of BMI on RBD in PD patients.

**Figure 4:**
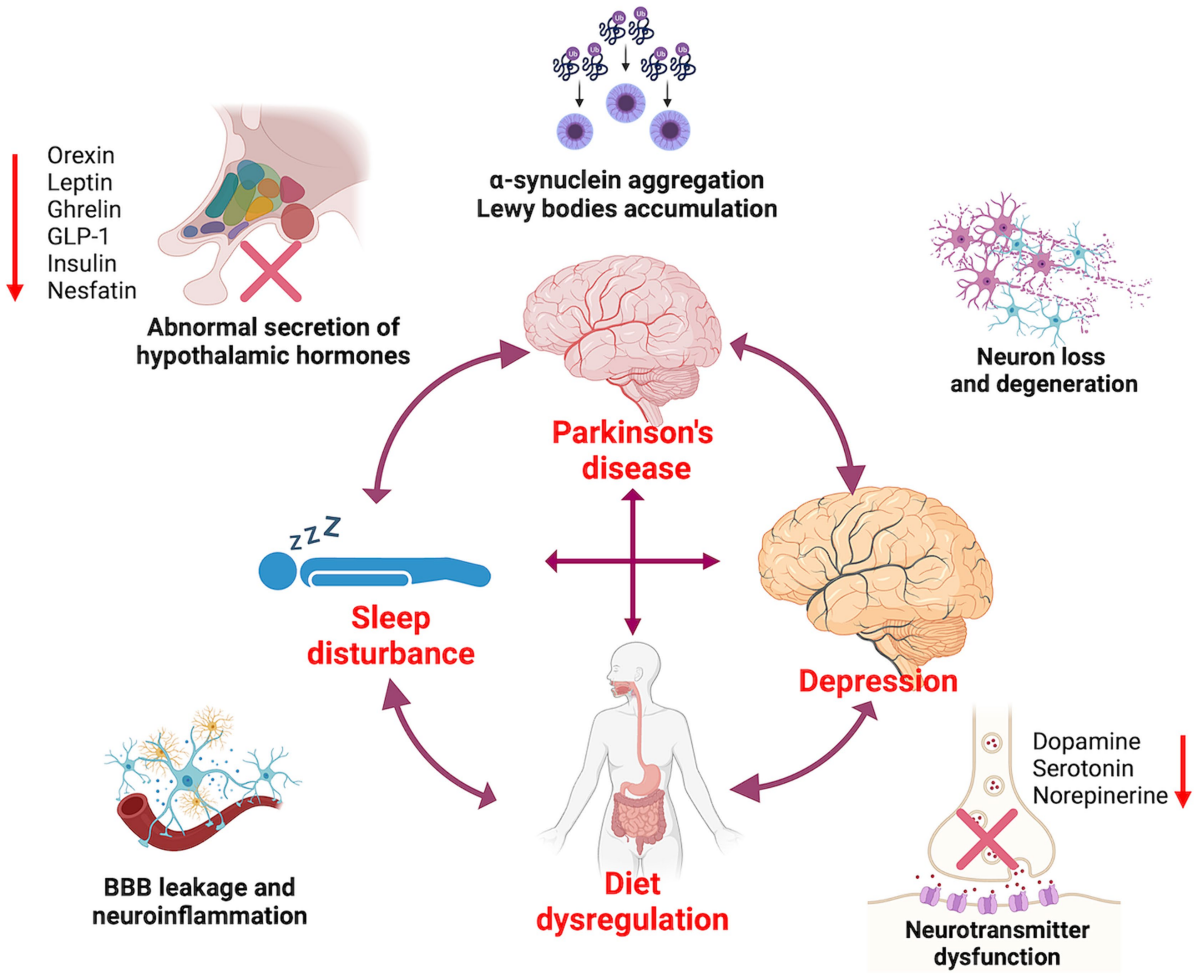
The interactions between Parkinson’s disease, diet dysregulation, sleep disorders, and depression.

In the RCS models, BMI tended to be positively associated with the odds of RBD up to change points with little association above these change points. And we also estimated an increased odds of RBD among obese individuals compared with those of healthy weight in PD. Although the study confirmed the nonlinear association between BMI and RBD in PD, the mechanisms did not seem straightforward. It has been reported that the pathology of PD originates from the gastrointestinal tract, which also serves as an energy portal, and develops upward along the neural pathway to the central nervous system, including the dorsal motor nucleus of substantia nigra, vagus, and hypothalamus. These areas are also involved in energy metabolism control and sleep regulation (30, 31). Some studies have suggested that altered sleep quality and circadian clock could directly affect neuro-endocrine systems involved in the regulation of energy balance, resulting in overweight. This includes the sympathetic nervous system, hypothalamic–pituitary–adrenal (HPA) axis, brain metabolism-related peptides such as glucagon-like peptide 1 (GLP-1), insulin, leptin, and ghrelin levels (32). Furthermore, it has been found that a lack of orexin or insufficient orexin signaling, along with low physical activity, might promote the coexistence of sleep disorders and overweight (33) (Figure 4). Conversely, RBD itself might predispose individuals to worsening overweight due to sleep deprivation, increased sympathetic activation, sleep fragmentation, ineffective sleep, and insulin resistance. This could potentially lead to diabetes and aggravation of obesity (34, 35) (Figure 4). Thus, it seems that RBD and weight status in the context of PD form a cycle interrelating with each other. It is crucial to understand the precise interaction between them.

Subgroup analyses showed strong interactions of depression and the clear heterogeneity in the associations of BMI and RBD, meaning that the association of RBD with BMI would be affected by the mood status of PD patients. The odds of RBD were higher for obese individuals with depression compared to those without depression, which were partly consistent with results from some major studies.

It has been shown that patients with depression exhibit abnormalities in sleep parameters throughout all phases of sleep architecture. Among these alterations, changes in REM sleep are particularly prominent and are often considered a distinct biological marker of depression (36). Patients with depression may experience longer disease duration, more severe symptoms, and a higher overall illness severity compared to individuals without depression. As a result, they are at higher odds of developing RBD (37). Several studies investigating the pathophysiological mechanisms underlying the relationship between sleep disturbance and depression have found that dysregulation of monoamine neurotransmitters such as serotonin, norepinephrine, and dopamine, which are responsible for REM sleep abnormalities, is also associated with the presentation of depression (38, 39). Furthermore, sleep disorders can increase markers of inflammation by activating the sympathetic nervous system and β-adrenergic signaling, leading to increased NF-κB activity and activation of inflammatory gene expression. Notably, there is a strong association between inflammation and depression (40, 41) (Figure 4). To better understand the link between sleep and depression, further research is needed to elucidate the role of inflammation, monoamines, and other related neurotransmitters.

This study might provide more insight into the complex interactions between PD, diet dysregulation, sleep disorders, and depression in clinical practice. Conducting a comprehensive assessment, including evaluating PD symptoms, sleep disorders, BMI, and depression during diagnosis and treatment might be essential. In addition, the multidisciplinary management approach including neurologist, primary care physician, psychologists, and nutritionists is crucial for PD patients. Furthermore, the combination of pharmacological interventions (anti-parkinsonism meditations, hypnotics and antidepressants), non-pharmacological therapies (such as cognitive-behavioral therapy for insomnia or light therapy for circadian rhythm disorders), and lifestyle modifications (such as regular physical exercise and well-rounded nutritional diet) should be highlighted. The better understanding of the pathophysiological mechanisms between PD, diet regulation, sleep and mood is urgently needed for comprehensive manage this comorbidity.

There were several limitations to this study. First, despite efforts to adjust for confounding variables leading to potential confounding bias in the current study, bias might still be inherent and sometimes inevitable in cross-sectional studies. One reason is the reliance on data collected at a single point in time, limiting researchers’ ability to establish temporal relationships or causality between variables. Additionally, cross-sectional studies often rely on self-reported data, which can be subject to recall bias or social desirability bias. Selection bias might arise from the non-random sampling of participants, resulting in an unrepresentative sample and limit the generalizability of findings to the target population (42–44). Thus, prospective and large cohort studies are still needed in the future. Second, although we tried to adjust many variables that can be related to RBD in PD, factors such as smoking and drinking could not be include due to the restriction of data source in PPMI. Third, in our participants, the number of patients with BMI <18·5 kg/m^2^ was relatively small, which should be considered when interpreting results.

## 5 Conclusion

In conclusion, this study demonstrated a significant dose–response association between BMI and RBD in PD patients, even after adjusting for potential confounders. And there was a depression-based difference in the impact of BMI on RBD. Regular monitoring of BMI is thus important for patients with PD, particularly patients with depression. Future randomized controlled trials or cohort studies are needed to validate these findings and provide more precise prevention and treatment options for RBD in PD.

## Data availability statement

The datasets presented in this study can be found in online repositories. The names of the repository/repositories and accession number(s) can be found at: www.ppmi-info.org/data.

## Ethics statement

The studies involving humans were approved by the Institutional Review Board at each site (the full list is available as a Supplementary File). The studies were conducted in accordance with the local legislation and institutional requirements. The participants provided their written informed consent to participate in this study.

## Author contributions

S-CG: Conceptualization, Data curation, Formal analysis, Funding acquisition, Software, Writing – original draft, Writing – review & editing. X-LY: Conceptualization, Data curation, Formal analysis, Funding acquisition, Writing – original draft, Writing – review & editing. PY: Conceptualization, Data curation, Formal analysis, Writing – original draft, Writing – review & editing. Y-YL: Data curation, Investigation, Writing – review & editing. C-DW: Data curation, Investigation, Writing – review & editing. M-JG: Data curation, Investigation, Writing – review & editing. L-MX: Data curation, Investigation, Writing – review & editing. CG: Data curation, Investigation, Writing – review & editing. YW: Data curation, Investigation, Writing – review & editing. Y-QH: Data curation, Investigation, Writing – review & editing. C-XY: Data curation, Investigation, Writing – review & editing. YC: Conceptualization, Data curation, Formal analysis, Writing – original draft, Writing – review & editing. QY: Conceptualization, Funding acquisition, Writing – original draft, Writing – review & editing.

## Footnotes

^1^ www.ppmi-info.org/data

## Glossary

BMI: body mass index
REM: rapid eye movement
RBD: rapid eye movement sleep behaviour disorder
RBDSQ: rapid eye movement sleep behaviour disorder screening questionnaire
PD: Parkinson’s disease
PPMI: Parkinson’s progression markers initiative
RCS: restricted cubic spline
SWEDD: scans without evidence of dopaminergic deficit
MDS: movement disorders society
LEDD: levodopa equivalent daily dose
UPDRS: unified Parkinson’s disease rating scale
SCOPA-AUT: scale for outcomes for Parkinson’s disease-autonomic function
STAI: state-trait anxiety inventory
MSEADL: modified Schwab and England activities of daily living scale
CSF: cerebrospinal fluid
TD: tremor dominate
PIGD: postural instability/gait disorder
MOCA: Montreal cognitive assessment
GDS-15: 15-item geriatric depression scale
SD: standard deviation
IQR: interquartile range
CI: confidence interval
