# Supplementary material for: Association of body mass index with rapid eye movement sleep behavior disorder in Parkinson’s disease
Source: Front Neurol. 2024 May 23;15:1388131. doi: 10.3389/fneur.2024.1388131 (PMC11155480; doi:10.3389/fneur.2024.1388131)
Supplement: Supplementary file 1 [file Data_Sheet_1.docx]

e-Table 1. Effect modification by motor subtype, sex, hypertension, or depression on the association between BMI and RBD in PD

| Model | Interaction | βvalue | P value |
| --- | --- | --- | --- |
| Model A | BMI^2^*motor subtype | 0.001 | 0.11 |
| Model A | BMI*motor subtype | -0.06 | 0.34 |
| Model B | BMI*motor subtype | 0.03 | 0.23 |
| Model A | BMI^2^*sex | 0.05 | 0.53 |
| Model A | BMI*sex | -0.001 | 0.29 |
| Model B | BMI*sex | 0.03 | 0.27 |
| Model A | BMI^2^*hypertension | 0.01 | 0.33 |
| Model A | BMI*hypertension | -0.002 | 0.44 |
| Model B | BMI*hypertension | 0.03 | 0.17 |
| Model A | BMI^2^*depression | 0.003 | 0.03 |
| Model A | BMI* depression | -0.09 | 0.03 |

Abbreviations: BMI, body mass index; PD, Parkinson’s disease; RBD, rapid eye movement sleep behavior disorder.


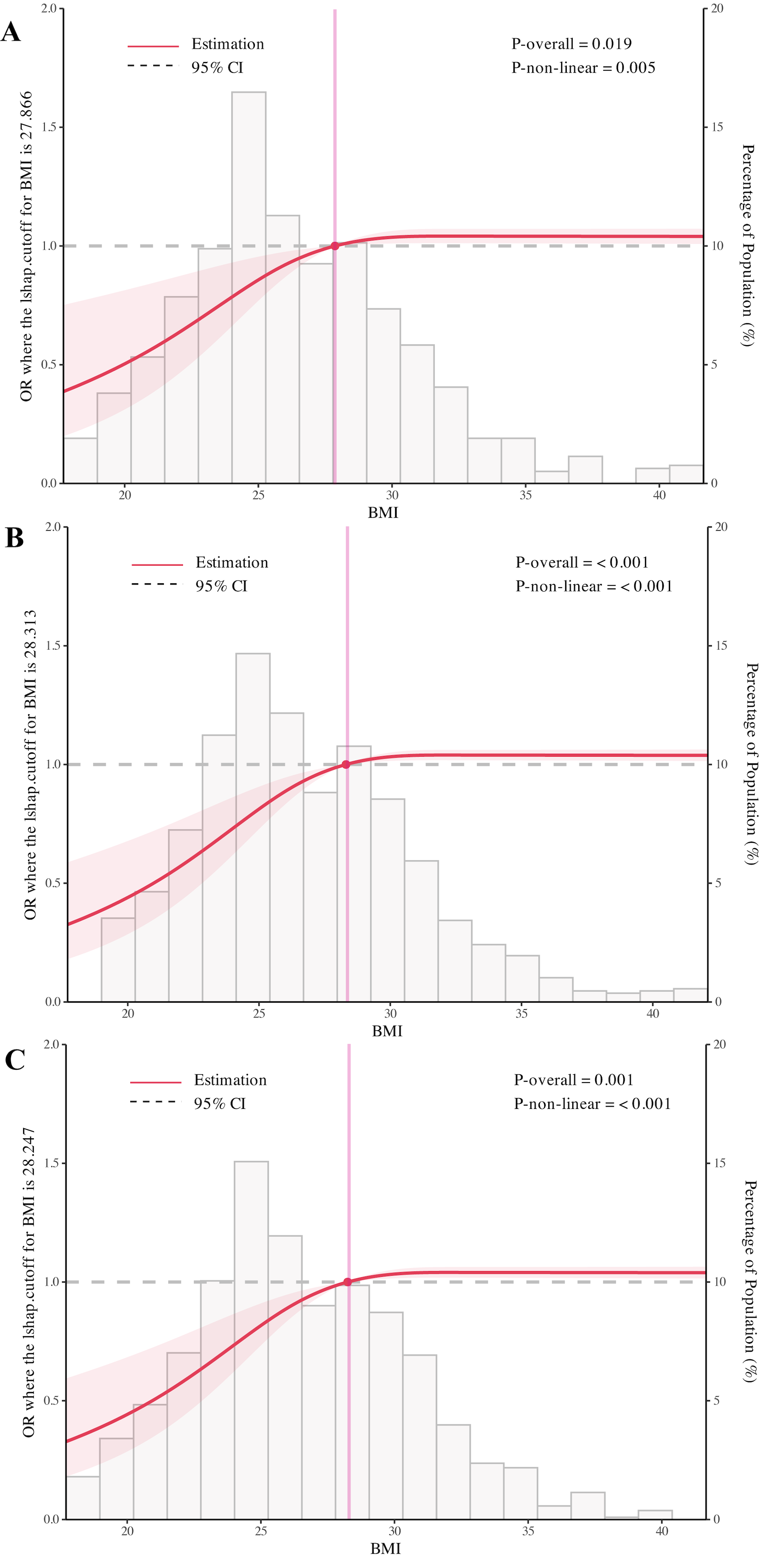


e-Figure 1. Sensitivity analyses on the association of Body Mass Index with rapid eye movement sleep behavior disorder in Parkinson’s Disease. (A) The association of Body Mass Index with rapid eye movement sleep behavior disorder in Parkinson’s Disease among participants withMontreal Cognitive Assessment score ≥ 26; (B) The association of Body Mass Index with rapid eye movement sleep

behavior disorder in Parkinson’s Disease among participants in the first and second categories of education; (C) The association of Body Mass Index with rapid eye movement sleep behavior disorder in Parkinson’s Disease after excluding other ethnic groups. All figures were created with the use of R software (version 3.3.3, <https://www.r-project.org/>).
