# Supplementary material for: Association of body mass index with rapid eye movement sleep behavior disorder in Parkinson’s disease
Source: Front Neurol. 2024 May 23;15:1388131. doi: 10.3389/fneur.2024.1388131 (PMC11155480; doi:10.3389/fneur.2024.1388131)
Supplement: Supplementary file 2 [file Data_Sheet_2.docx]

**Institutional Review Boards of PPMI:**

David Russell, MD, PhD, Laura Leary, BS, Institute for Neurodegenerative Disorders, New Haven, CT; Stewart Factor, DO, Barbara Sommerfeld, RN, MSN, Emory University of Medicine, Atlanta, GA; Penelope Hogarth, MD, Emily Pighetti, Oregon Health and Science University, Portland, OR; Karen Williams, Northwestern University, Chicago, IL; David Standaert, MD, PhD, Stephanie Guthrie, University of Alabama at Birmingham; Robert Hauser, MD, Holly Delgado, RN, University of South Florida, Tampa, FL; Joseph Jankovic, MD, Christine Hunter, RN, CCRC, Baylor College of Medicine, Houston, TX; Matthew Stern, MD, Baochan Tran, University of Pennsylvania, Philadelphia, PA; Jim Leverenz, MD, Marne Baca, University of Washington, Seattle, WA; Sam Frank, MD, Cathi-Ann Thomas, RN, MS, Boston University, Boston, MA; Irene Richard, MD, Cheryl Deeley, MS, RNC, University of Rochester, Rochester, NY; Linda Rees, The Parkinson’s Institute, Sunnyvale, CA; Fabienne Sprenger, Innsbruck Medical University, Innsbruck, Austria; Elisabeth Lang, Paracelsus-Elena Klinik, Kassel, Germany; Holly Shill, MD, Sanja Obradov, BA, Banner Research Institute, Sun City, AZ; Hubert Fernandez, MD, Adrienna Winters, BS, Cleveland Clinic, Cleveland, OH; Daniela Berg, MD, Katharina Gauss, University of Tuebingen, Germany; Douglas Galasko, MD, Deborah Fontaine, RNCS, MS, University of California, San Diego; Zoltan Mari, MD, Melissa Gerstenhaber, RNC, MSN, Johns Hopkins University, Baltimore, MD; David Brooks, MD, Sophie Malloy, MD, Imperial College London, UK; Paolo Barone, MD, PhD, Katia Longo, MD, Universita Federico II, Naples, Italy.

**Reference:**

Parkinson Progression Marker Initiative. The Parkinson Progression Marker Initiative (PPMI). Prog Neurobiol. 2011 Dec;95(4):629-35. doi: 10.1016/j.pneurobio.2011.09.005. Epub 2011 Sep 14. PMID: 21930184; PMCID: PMC9014725.
